# A novel missense variant in *TRAPPC2* causes X-linked spondyloepiphyseal dysplasia tarda: A case report

**DOI:** 10.1097/MD.0000000000025169

**Published:** 2021-03-19

**Authors:** Li Zhang, Jinling Wang, Guanping Dong, Dingwen Wu, Wei Wu

**Affiliations:** a Department of Endocrinology; b Department of Genetics and Metabolism, The Children's Hospital, Zhejiang University School of Medicine, National Clinical Research Center for Child Health, Hangzhou, China.

**Keywords:** growth hormone, short stature, skeletal dysplasia, *TRAPPC2*, X-linked spondyloepiphyseal eysplasiat tarda

## Abstract

**Rationale:**

X-linked spondyloepiphyseal dysplasia tarda (X-linked SEDT) is a rare hereditary cause in childhood short stature due to mutations in trafficking protein particle complex subunit 2 *(TRAPPC2)* gene located on chromosome Xp22. Several pathogenic variants in *TRAPPC2* have been reported, but missense variants are rare.

**Patient concerns:**

A 13-year, 8-month-old Chinese Han boy presenting with short stature for the past 7 years.

**Diagnosis:**

X-linked SEDT was established by a combination of clinical and radiographic features, confirmed by targeted next-generation sequencing. Genetic testing of the *TRAPPC2* gene revealed a novel missense variant with c.260A>C (p.H87P) hemizygote in exon5. The mother was found to be a heterozygous *TRAPPC2* carrier, whereas the father was normal.

**Interventions:**

Patient was treated with recombinant human growth hormone daily. Patient's height, glucose level, and possible progressive joint and back pain with osteoarthritis were under intensive observation regularly.

**Outcomes:**

The patient achieved 2.1 cm height gain over the first 3 months’ recombinant human growth hormone treatment without joint or back pain. However, the therapy was terminated because of increased glucose level on follow-up.

**Lessons:**

The short stature is a noteworthy problem for X-linked SEDT cases. We report a novel missense variant site in *TRAPPC2* treated with growth hormone in the literature. We do not recommend the use of recombinant human growth hormone on patients with X-linked SEDT for the concern of glucose homeostasis.

## 1 Introduction

X-linked spondyloepiphyseal dysplasia tarda (X-linked SEDT) is an X-linked skeletal dysplasia typically affecting male subjects.^[1]^ X-linked SEDT is a rare cause of childhood short stature characterized by defective structures of vertebral bodies and/or of epiphyses of the long bones, leading to short trunk and precocious osteoarthritis.^[2]^ Trafficking protein particle complex subunit 2 *(TRAPPC2)* is the causative gene, encoding a 140-amino acid protein called transport protein particle complex subunit 2 (TRAPPC2). Fifty-eight pathogenic variants in *TRAPPC2* have been reported including 32 insertions or deletions, 10 splice site variants, 9 nonsense variants. Nevertheless, reported missense variants are relatively rare including c.139G>T (p.Asp47Tyr), c.218C>T (p.Ser73Leu), c.239A>G (p.His80Arg), c.248T>C (p.Phe83Ser), c.389T>A (p.Val130Asp).^[3]^

X-linked SEDT patients exhibit almost identical phenotype irrespective of *TRAPPC2* pathogenic types in various ethnic groups thus far. The current management for X-linked SEDT involves surgical intervention for bone dysplasia, chronic pain management, and regular surveillance. However, there is no method reported to relieve the growth retardation until now.^[4]^

In the study, we report a novel missense variant in *TRAPPC2* causing X-linked SEDT in a Chinese boy by targeted next-generation sequencing. To the best of our knowledge, this was the first X-linked SEDT case treated with recombined growth hormone therapy with surveillance.

## 2 Case report

A 13-year, 8-month-old Chinese Han boy was referred to endocrinology department, presenting with short stature for the last 7 years. He was an otherwise healthy boy with normal birth weight and birth length after an uneventful pregnancy. No subjective symptoms were reported, including back or joint pain, or scoliosis. His motor and cognitive functions were normal. Upon physical examination, his height was 126.4 cm (-4SD), sitting height 70.5 cm, and weight 22.5 Kg. He was disproportionately short stature with a relatively short trunk and barrel chest. Arm span 138.0 cm, typically exceeded height by 11 cm. The patient had no dysmorphic facial features with Tanner stage 2. No other family members had short stature (short trunk or abnormal arm span), or a history of degenerative joint disease or hip joint replacement or other medical issues (Supplementary Figure 1).

Serum concentration of insulin-like growth factor 1 and thyroid hormone was normal. The growth hormone stimulation tests (L-dopa and arginine) for short stature excluded the growth hormone deficiency with peak growth hormone level 22.6 ng/ml (Table 1).

**Table 1:** The clinical evaluation of the X-linked SEDT case.

	Before rhGH	On rhGH 3 mo	off rhGH 6 mo	Normal reference
Peak GH	22.6	NA	NA	GHD: <10.0
IGF-1 (ng/mL)	204.0	233.0	225	143-693
Bone age	11Y2M	11Y4M	11Y4M	G-P BA assessment
peak LH (IU/L)	18.7	NA	NA	In puberty: LH _peak_> 5 LH _peak_/FSH_peak_>0.6
peak FSH (IU/L)	12.1	NA	NA	
Basal LH (IU/L)	0.39	0.69	0.65	<2.8
Basal FSH (IU/L)	0.39	3.70	2.8	1.2–7.0
Testosterone (nmol/L)	<0.69	<0.69	<0.69	0.52–27.74
TSH (MIU/L)	2.373	3.135	2.855	0.35–4.94
Total thyroids (nmol/L)	136.59	109.66	123.45	62.68–150.8
Phosphonate (mmol/L)	1.36	1.53	1.54	1.29–2.26
Total calcium (mmol/L)	2.55	2.53	2.55	2.20–2.65
Vitamin D (nmol/L)	51.4	55.7	54.6	27.5-150

FSH = follicle stimulating hormone, GH = growth hormone, IGF-1 = insulin-like growth factor-1, LH = luteinizing hormone, NA = not available, TSH = thyroid stimulating hormone.

**Figure 1:**
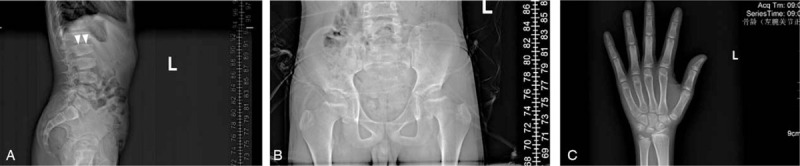
Radiographs of the X-linked SEDT case.

Radiographs revealed lumbosacral platyspondyly with characteristic superior and inferior humping of the vertebral bodies seen on lateral view (Fig. 1A). The pelvis was somewhat narrow, and the femoral necks were short (Fig. 1B). Osteoarthritic changes such as joint space narrowing, extensive osteophytes, were not observed in all involved joints including shoulders, wrists, and knees. His bone age (BA) was 11-year, 2-month old estimated by G-P BA assessment, extremely younger than chronological age (Fig. 1C).

For genetic analysis, blood samples were obtained with written informed consent from the patient and parents. Genetic analysis was also approved by the Medical Ethics Committee of the Children's Hospital of Zhejiang University School of Medicine. The genomic DNA of the patient and his parents was isolated from 200 μL, peripheral blood samples using QIAampDNABlood Mini Kit (Qiagen GmbH, Hilden, Germany). Qubit dsDNA detection kit and Qubit 4 fluorometer (Invitrogen, Carlsbad, CA) were used to detect DNA concentration and purity. An adapter-ligated library was produced with Agilent Sure Select Target Enrichment System (Agilent Technologies Inc, Santa Clara, CA) according to the manufacturer's instructions. The capture library was performed using an XT Inherited Disease Panel (cat No. 5190–7519, Agilent Technologies Inc) containing 2742 genes. Clusters were then generated by isothermal bridge amplification using an Illumina cBot Station, and sequencing was performed on an Illumina HiSeq 2500 System (Illumina Inc, San Diego, CA). Using human genome hg19 as the reference, alignment of sequence and repeated labeling were performed using BWM 0.7.17 and Picard bioinformatics software version 2.5.0 for biological analysis and interpretation. GATK 4.0.0.0 and Samtools 1.8 were used to identify mutation sites.

Targeted next-generation sequencing revealed a novel missense variant c.260A>C in the *TRAPPC2* exon5 localized to ChromosomeXp22 in the proband, causing p. His87Pro mutation. His mother was found to be heterozygous for the c.260A>C substitution, confirming her carrier status, whereas the father was found to be normal (Supplementary Figure 2). Based on the ACMG criteria, the novel c.260A>C in the *TRAPPC2* gene was the pathogenic variant of this X-linked SEDT family.

Based on the clinical manifestation, radiographic and genetic findings, the case was diagnosed as X-linked SEDT caused by a novel maternally inherited missense variant c.260A>C (p.His87Pro) in the *TRAPPC2* gene.

With family's strong desire to improve the height loss, the patient was imitated with 0.06 mg/Kg/d (i.e., 0.17iu/Kg/d) of recombinant human growth hormone (rhGH) after exclusion of contraindications including blood glucose level and pituitary MRI. Written informed consent from parents and patient was obtained. He achieved 2.1 cm height gain over the first 3-month treatment with standing height 128.5 cm, sitting height 71.0 cm. The patient was in Tanner stage 2 with almost the same BA and basal luteinizing hormone as well as basal follicle-stimulating hormone level as 3 months ago (Table 1). No joint pain and scoliosis were reported during the intensive follow-up.

**Figure 2:**
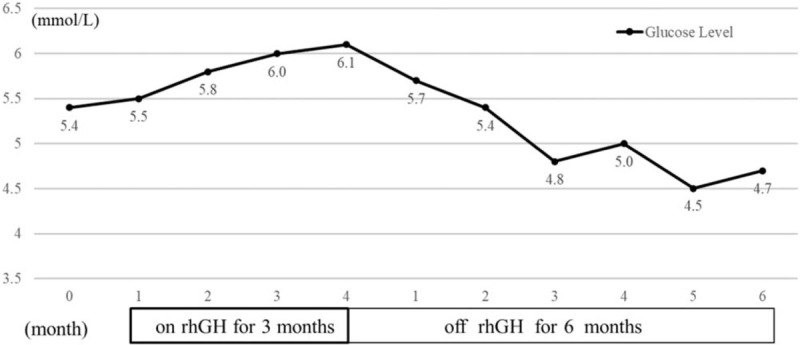
The rhGH treatment and surveillance.

However, the routine blood glucose test revealed a slight increase (morning fasting glucose 6.1 mmol/L) after 3 months’ rhGH therapy. The rhGH injection was terminated and his blood glucose monitoring went on. Patient's fasting glucose fluctuated between 4.5 and 5.7mmol/L over the next 6 months, with HbA1c 4.6% (Fig. 2). His standing height was 129.0 cm after 6-month follow-up. No other abnormal biochemical results were reported (Table 1).

## 3 Discussions

X-linked SEDT is a rare cause of childhood short stature and skeletal dysplasia with an estimated prevalence of 1.7 per 1,000,000 individuals.^[5]^ Here we present a 13-year-old boy with typical features of X-linked SEDT, including disproportionate short stature, delayed linear growth beginning around 6 to 8 years of age, and flattening of the vertebrate body with characteristic humping. Targeted next-generation sequencing revealed a novel missense variant c.260A>C in the *TRAPPC2*, confirming the diagnosis.

**Figure 3:**
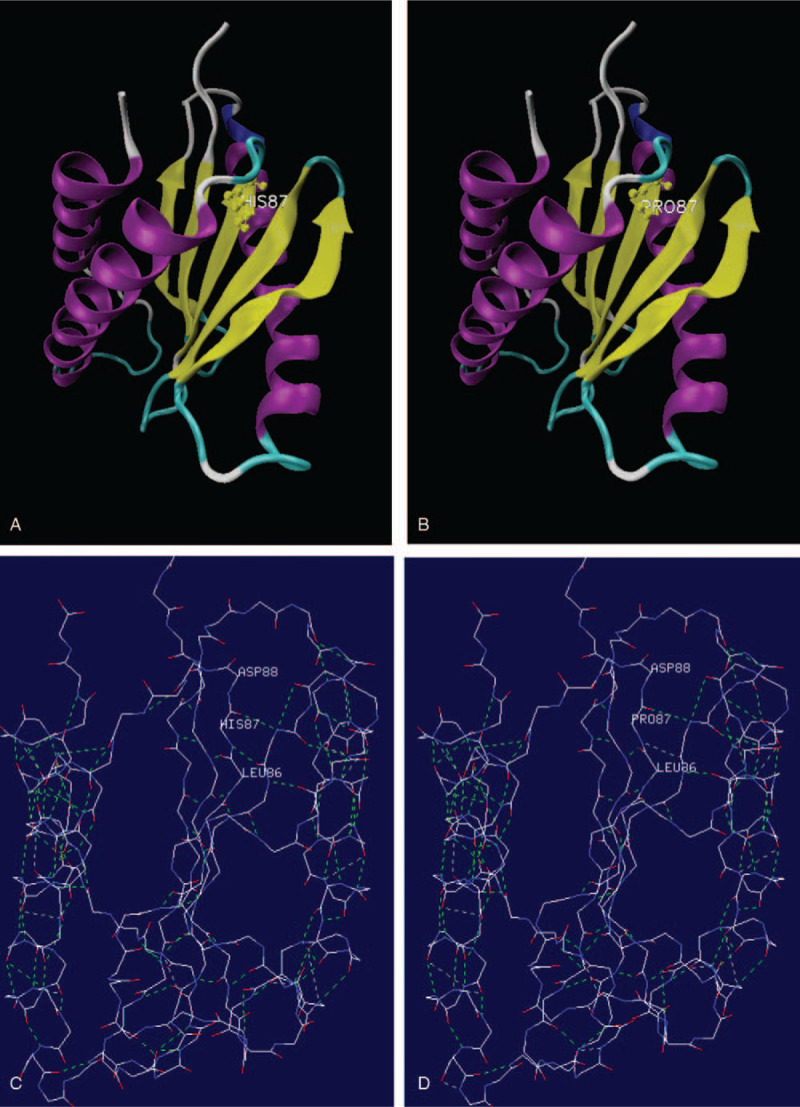
The predicted structure of the mutated TRAPPC2 protein (p. His87Pro).

The human *TRAPPC2* gene consists of 6 exons organized from exon 3 to exon 6, on which only 5 missense *TRAPPC2* variants have been reported to date.^[6]^ Our discovery c.260A>C on *TRAPPC2* gene exon5 results in a novel missense mutation in protein translation (His87Pro). The simulation structure of the mutated TRAPPC2 protein is presented in Figure 3 with SWISS-model,^[7]^ which is predicted to be highly damaging using different *in silico* software (SIFT, Polyphen2, MutationTaster, REVEL). The novel variant found in this study is adjacent to 2 reported pathogenic variants c.239A>G (p. His80Arg) and c.248T>C (p. Phe83Ser).^[8,9]^ They were predicted to misfold and affect the overall structure of TRAPPC2 protein.^[10]^ It is proposed that loss function of TRAPPC2 protein might affect the Golgi integrity or the collagen trafficking,^[11,12]^ causing a defect in the secretion of extracellular matrix proteins by chondrocytes or dissociation from the membrane.^[1,13]^ However, further studies are required to understand the molecular mechanisms of pathophysiology between the probable pathogenic c.260A>C variant and X-linked SEDT.

Studies demonstrate that all individuals with TRAPPC2 variants suffer from X-linked SEDT.^[1,6]^ For genetic counseling, 2 family members (III-1 and III-2) in child-birth age underwent the targeted next-generation sequencing^[14]^ and both were negative, ruling out the possibility of having affected children. X-linked SEDT adults suffer from progressive joint and back pains, while teenagers exhibit retarded linear growth beginning around the age of 6 to 8 years.^[1]^

At present, no specific treatments for the severe height loss of X-linked SEDT cases are available worldwide. A few studies show that growth hormone therapy has a beneficial effect on skeletal dysplasia patients with severe short stature (-3 SD to -4 SD), such as tricho-rhino-phalangeal syndromes and pycnodysostosis.^[15–17]^ Our X-linked SEDT patient achieved inspiring 2.1 cm height gain in the 3-month rhGH treatment with a dosage equivalent to idiopathic short stature patients. However, the efficacy of rhGH is not confirmed, and we cannot rule out the effect of pubertal growth spurt, although with no BA or luteinizing hormone/follicle-stimulating hormone progress. GH treatment seems to give better results when administered at puberty for hypochondroplasia patients with short stature.^[4]^ But no studies involved the relationships between pubertal development and growth hormone for short stature on X-linked SEDT cases.^[4,16]^

Moreover, glucose homeostasis is a big concern because glucose level increased in the routine monitoring and returned to normal after rhGH termination over 6 months. X-linked SEDT patients are not reported with abnormal glucose level or diabetes previously. We speculate that X-linked SEDT patients are more prone to be hyperglycemic with rhGH treatment.^[18,19]^ Thus, we do not recommend X-linked SEDT patients with rhGH therapy for safety, although our patient and parents expressed their strong willingness to restart rhGH treatment.

In conclusion, this is the first report of a novel missense variant c.260A>C (p. His87Pro) in *TRAPPC2* gene causing severe short stature in an X-linked SEDT Chinese boy, expanding the genetic spectrum of skeletal dysplasia. We do not recommend rhGH therapy for glucose homeostasis.

## Author contributions

**Conceptualization:** Wei Wu.

**Formal analysis:** Li Zhang.

**Funding acquisition:** Jinling Wang, Guanping Dong, Wei Wu.

**Investigation:** Li Zhang, Jinling Wang.

**Methodology:** Dingwen Wu.

**Project administration:** Guanping Dong.

**Resources:** Li Zhang.

**Software:** Dingwen Wu.

**Supervision:** Wei Wu.

**Writing – original draft:** Li Zhang, Jinling Wang.

**Writing – review & editing:** Li Zhang, Jinling Wang, Guanping Dong, Wei Wu.

## Supplementary Material

Supplemental Digital Content

## Supplementary Material

Supplemental Digital Content
