# Supplementary material for: A novel missense variant in TRAPPC2 causes X-linked spondyloepiphyseal dysplasia tarda: A case report
Source: Medicine (Baltimore). 2021 Mar 19;100(11):e25169. doi: 10.1097/MD.0000000000025169 (PMC7982231; doi:10.1097/MD.0000000000025169)
Supplement: Supplemental Digital Content [file medi-100-e25169-s001.docx]

**Supplementary Figure1 The pedigree of the family with X-linked SEDT.**


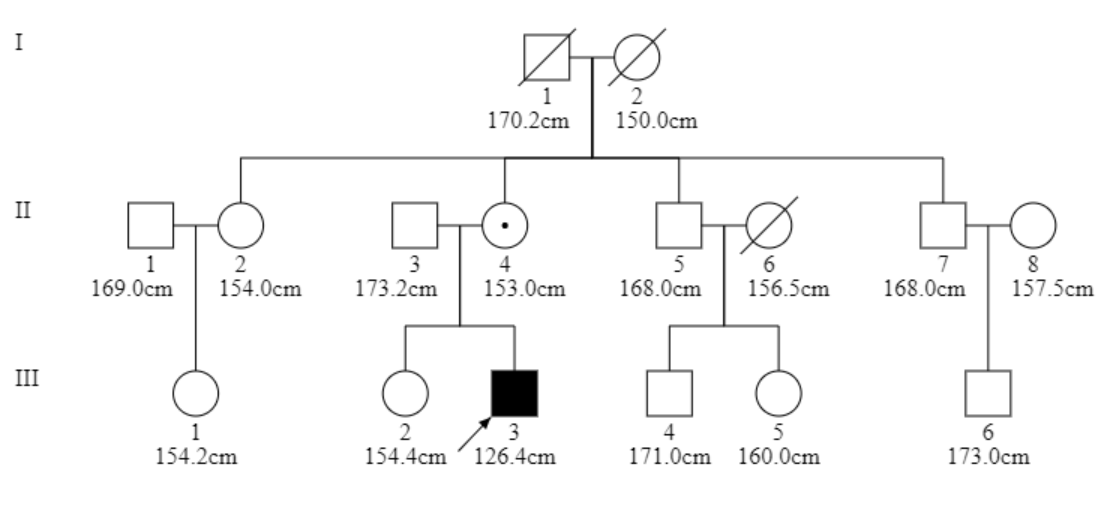


Black symbols denote affected subjects. The arrow indicates the proband (III 302). A circle with a dot in the middle indicates the status of carrier. All open boxes represent healthy males and open circles represent healthy females. Boxes or circles with a diagonal line indicate that the person has already died.
