# Supplementary material for: A novel missense variant in TRAPPC2 causes X-linked spondyloepiphyseal dysplasia tarda: A case report
Source: Medicine (Baltimore). 2021 Mar 19;100(11):e25169. doi: 10.1097/MD.0000000000025169 (PMC7982231; doi:10.1097/MD.0000000000025169)
Supplement: Supplemental Digital Content [file medi-100-e25169-s002.docx]

**Supplementary Figure2 Genetic sequencing results of the X-linked SEDT family.**

A novel missense variant c.260A>C（p.His87Pro） in the *TRAPPC2* gene was identified and illustrated in the schematic diagram.
